# Gait Speed and Gait Variability Are Associated with Different Functional Brain Networks

**DOI:** 10.3389/fnagi.2017.00390

**Published:** 2017-11-29

**Authors:** On-Yee Lo, Mark A. Halko, Junhong Zhou, Rachel Harrison, Lewis A. Lipsitz, Brad Manor

**Affiliations:** ^1^Institute for Aging Research, Hebrew SeniorLife, Boston, MA, United States; ^2^Harvard Medical School, Harvard University, Boston, MA, United States; ^3^Division of Gerontology, Department of Medicine, Beth Israel Deaconess Medical Center, Harvard Medical School, Harvard University, Boston, MA, United States; ^4^Berenson-Allen Center for Noninvasive Brain Stimulation, Beth Israel Deaconess Medical Center, Harvard Medical School, Harvard University, Boston, MA, United States; ^5^Department of Neurology, Beth Israel Deaconess Medical Center, Harvard Medical School, Harvard University, Boston, MA, United States

**Keywords:** gait, resting-state fMRI, functional connectivity, gait speed, gait variability

## Abstract

Gait speed and gait variability are clinically meaningful markers of locomotor control that are suspected to be regulated by multiple supraspinal control mechanisms. The purpose of this study was to evaluate the relationships between these gait parameters and the functional connectivity of brain networks in functionally limited older adults. Twelve older adults with mild-to-moderate cognition “executive” dysfunction and relatively slow gait, yet free from neurological diseases, completed a gait assessment and a resting-state fMRI. Gait speed and variability were associated with the strength of functional connectivity of different brain networks. Those with faster gait speed had stronger functional connectivity *within* the frontoparietal control network (*R = 0.61, p = 0.04*). Those with less gait variability (i.e., steadier walking patterns) exhibited stronger *negative* functional connectivity *between* the dorsal attention network and the default network (*R = 0.78, p < 0.01*). No other significant relationships between gait metrics and the strength of within- or between- network functional connectivity was observed. Results of this pilot study warrant further investigation to confirm that gait speed and variability are linked to different brain networks in vulnerable older adults.

## Introduction

Age-related decline in locomotor control often leads to falls and adversely affects one’s quality of life and independence. Locomotor control is most commonly assessed by measuring average preferred gait speed and/or gait variability (i.e., the degree of steadiness about the average of a given stride parameter over consecutive strides). Intriguingly, these two metrics are often uncorrelated (Hollman et al., 2011; Lord et al., 2013) and may be independently influenced by experimental stressors (Hausdorff, 2005, 2007). It seems reasonable to hypothesize, therefore, that gait speed and gait variability may be regulated by fundamentally different functional networks within the brain.

The relationships between metrics of gait and brain function *during walking* have been challenging to establish primarily because current neuroimaging tools are sensitive to head and body movements (Hamacher et al., 2015; Wittenberg et al., 2017). Alternatively, resting-state functional magnetic resonance imaging (rs-fMRI) is a powerful tool that enables estimation of functional organization within the brain (Biswal et al., 1995; van den Heuvel and Pol, 2010) and subsequently, determination of how this organization is linked with function and behavior (Lee et al., 2013; Cruz-Gómez et al., 2014; Connolly et al., 2016). Rs-fMRI can be used to identify highly replicable functional networks (Fox and Raichle, 2007; Yeo et al., 2011) and to quantify the patterns of functional connectivity within and between networks (Yeo et al., 2011), providing a reliable and measureable tool to assess cortico-cortical connectivity and its link with complicated human behaviors such as gait (Yuan et al., 2015). Moreover, as rs-fMRI is a “task-free” tool, it minimizes physical movements and avoids confounding from unrelated cortical processes present during the execution of a given task (Yeo et al., 2011).

Recent studies have demonstrated that slow gait speed associates with alterations in the function of the frontoparietal control network (Yuan et al., 2015; Jor’dan et al., 2017) – a network closely linked to executive function. Yuan et al. (2015) reported that functional connectivity within a cluster of frontal and parietal regions was related to gait speed in healthy adults; however, they did not report on the strength or direction of this relationship. Moreover, no studies to date have used rs-fMRI to establish links between fundamentally different properties of gait (i.e., speed and variability) and the functional connectivity of established brain networks. The objective of this study was thus to establish the relationship between clinically important measures of locomotor control and the strength of resting-state functional connectivity *within* and *between* functional brain networks in older adults. To accomplish this objective, we performed an analysis of an existing dataset collected from a small sample of ambulatory, non-demented older adults with mild-to-moderate cognitive-motor deficits. We hypothesized that gait speed and variability would be dependent upon distinct functional networks within the brain.

## Materials and Methods

We conducted a secondary analysis of baseline data from of a double-blinded, pilot randomized controlled trial on the effects of non-invasive brain stimulation on older adults. Inclusion criteria for that study included men and women who (1) were aged 65 years or older, (2) walked relatively slowly as indicated by a 4 m over-ground preferred walking speed of less than 1.0 m/s (Guralnik et al., 1995), and (3) exhibited mild-to-moderate cognitive “executive” dysfunction as indicated by a Trail Making Test (TMT) B time below the 25th percentile of age- and education-based norms (Tombaugh, 2004). The TMT test is considered as an index of executive function (Arbuthnott and Frank, 2000). In Part A, participants were asked to connect a series of numbers in sequential order on a sheet of paper as quickly and accurately as possible. In Part B, participants were asked to connect numbers or letters in alternating sequence (e.g., 1, A, 2, B, etc.). The time taken to complete each part was recorded. Participants were given up to 300 s to complete each part of the TMT test.

Participants were excluded if they (1) could not stand or ambulate unassisted, (2) had a clinical history of stroke, Parkinson’s disease, or other physician-diagnosed neurological disorders, (3) had a score of 18 or lower on the Mini-Mental State Examination (MMSE) (Folstein et al., 1975; Tombaugh and McIntyre, 1992) (to ensure that enrolled participants were able to understand and complete the study protocol), (4) had self-report of physician-diagnosed schizophrenia, bipolar disorder or other psychiatric illness, (5) had severe depressive symptoms as indicated by a Geriatric Depression Scale (GDS) score > 12 (van Marwijk et al., 1995), (6) had severe arthritis or lower-extremity pain, or (7) had physician-diagnosed peripheral neuropathy affecting the lower extremities.

Seventeen of 201 screened individuals were included in the parent study. Of these, 12 participants were eligible for and completed a baseline brain MRI scan and included in this analysis (Mean ±*SD*_age_ = 76.2 ± 9.5 years; 4 males and 8 females). The five participants who did not complete the MRI were ineligible due to the presence of potentially unsafe ocular implants.

All participants signed an informed consent form and the study was approved by the Hebrew SeniorLife Institutional Review Board.

### Data Acquisition and Analysis

Data analyzed in the current study were acquired during a screening visit, a baseline assessment and a functional MRI scan of the brain. Screening tests included MMSE, the TMT Parts A and B, and the Four Meter Walk Test (see inclusion and exclusion criteria above). Eligible participants then completed a gait assessment and resting-state fMRI measurement on two separate days separated by less than a week. Prior to obtaining a gait assessment, we also measured resting blood pressure and heart rate.

#### Gait Assessment

Participants completed an established protocol (Jor’dan et al., 2017), in which they performed one practice and five official trials of over-ground walking at preferred speed on a 60-foot oval indoor track with a 16-foot GAITRite mat placed along one side (CIR systems, Inc., Franklin, NJ, United States, 100 Hz sampling frequency). Participants walked approximately 1.25 times around the track such that they passed over the mat twice per trial. Across all participants, the fewest number of GAITRite-identified strides was 15. Previous reports have indicated that as few as 10 strides is sufficient for accurate estimation of both gait speed (Hollman et al., 2010) and stride time variability (Perera et al., 2016; Kroneberg et al., 2017). Participant instructions were as follows:

“*When I say go, walk across the mat and then continue walking until I tell you to stop. Walk at your normal speed, as if you were walking down the street to go to the store”*.

Average gait speed (m/s) and stride-to-stride time variability (%) were derived from each trial based upon concatenated footfalls from both passes over the mat. Gait speed was obtained by dividing the distance traveled (over the mat) by time. Gait variability (%) was defined as the coefficient of variation (CoV) about the mean right stride time. We chose to focus on stride time because stride time variability is reliable over time (Hausdorff, 2005; Brach et al., 2008) and sensitive to important health outcomes including falls in older adults (Brach et al., 2001; Hausdorff et al., 2001) and those with neurological disorders (Hausdorff et al., 1998; Sheridan et al., 2003; Balasubramanian et al., 2009). Each gait metric was averaged across the five trials for each participant. Participants were encouraged to rest between each walking trial to avoid potential fatigue. The GAITRite system has demonstrated high concurrent validity and test-retest reliability (McDonough et al., 2001; Bilney et al., 2003).

#### Resting-State MRI Acquisition and Analysis

Participants completed the MRI within a GE Signa HDxt 3 Tesla system with an 8-channel head coil within the Center for Advanced MR Imaging at the Beth Israel Deaconess Medical Center. Standard structural imaging was first acquired [MDEFT (Modified Driven Equilibrium Fourier Transform) sequence acquired axially with: 1.000 mm × 0.9375 mm × 0.9375 mm resolution; 6.616 ms TR, 2.84 ms TE; 15° flip angle; 1100 ms inversion time] followed by three 6-min runs of rs-fMRI BOLD sequences (3 mm × 3.75 mm × 3.75 mm, 3.2 s TR, 30 ms TE, 90° flip angle, 52 axial slices). Only two runs were available for three participants and in these cases, outcomes were derived from the two available runs. During the resting-state runs, participants were asked to fixate a cross within the MR bore for the entire duration of the resting run.

Resting-state fMRI were analyzed using a custom combination of software packages as previously described (Eldaief et al., 2011; Yeo et al., 2011; Halko et al., 2014). Acquired data were preprocessed with the following steps: spatial normalization to the MNI template, slice-time correction, motion-correction, and bandpass filtered for low frequency data (<0.1 Hz) spatial smoothing (7 mm FWHM). Ventricles, white matter and the global signal nuisance signals were regressed from the time-series.

**FIGURE 1:**
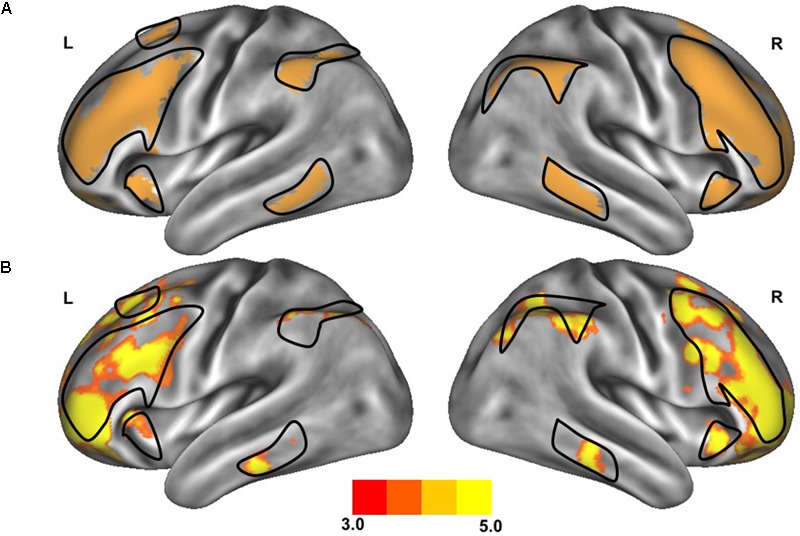
Resting-state functional connectivity of the frontoparietal control network. The standard map **(A)** of the frontoparietal control network derived from a large sample of healthy adults (Yeo et al., 2011) was used as a functional seed to determine the strength of functional connectivity within this network of the older adults with slow gait and executive dysfunction **(B)**. Warmer colors indicate stronger connectivity. The black outlined region represents the region selected for visualization of the voxel-wise analysis depicted in **Figures 4A,B**.

**FIGURE 2:**
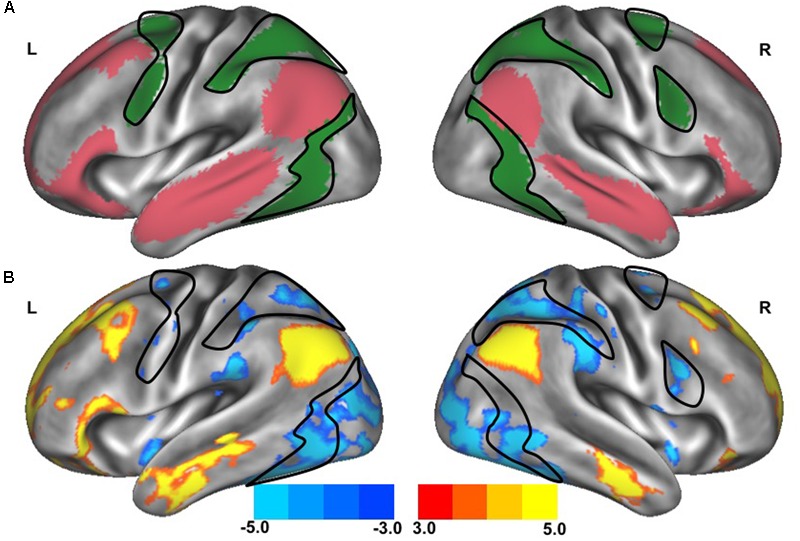
Resting-state functional connectivity between dorsal attention network and default network. The standard maps **(A)** of the dorsal attention network (green) and the default network (red) derived from a large sample of healthy young adults (Yeo et al., 2011) were used as functional seeds to determine the strength of functional connectivity *between* these networks of the older adults with slow gait and executive dysfunction **(B)**. Warmer colors represent regions with stronger *in-phase* functional connectivity to the default network; cooler colors represent regions with stronger *anti-phase* functional connectivity to the default network. The black outlined region represents the region selected for visualization of the voxel-wise analysis depicted in **Figures 4C,D**.

After preprocessing, seven networks were identified based on a previously defined parcellation from 1,000 brains (Yeo et al., 2011) and observed spontaneous activity within and between these seven networks. These seven highly replicated networks included: visual, somatomotor, limbic, dorsal attention, ventral attention, frontoparietal control, and default networks. These entire networks were selected as regions of interest to extract time-series. All functional connectivity measures were expressed as z-transformed Pearson correlation coefficients between time-series. For between-network connectivity (e.g., between dorsal attention network and default network), z-transformed Pearson correlation coefficients were computed between time-series from each of the network masks. For within-network connectivity, the mean z-transformed Pearson correlation coefficient was taken of the average time-series with each voxel’s time-series within the network mask. The strength of functional connectivity refers to the magnitude of Pearson correlation coefficients between the fMRI time-series among each spatial location. To create voxelwise maps of network connectivity, z-transformed Pearson correlation coefficients were computed for each voxel against the mean time-course from the network of interest. These maps were inspected to confirm that the spatial organization of these networks were similar to those observed in healthy controls, as can be observed in **Figures 1** and **2**. For display, these voxelwise maps were projected onto an average cortical surface within the Human Connectome Viewer.

### Statistical Analysis

Descriptive statistics were used to summarize participant characteristics and study outcomes including gait speed, gait variability and the strength of both *within-* and *between-*network functional connectivity. Bivariate analyses were used to test our primary hypothesis by determining the correlations between gait metrics and functional connectivity outcomes. Those functional connectivity variables that were significantly associated with gait outcomes were then entered into a regression model in order to adjust for participant age. The level of statistical significance for this proof-of-principle analysis was set at 0.05 after adjusting for age. Finally, secondary voxel-wise analyses were performed to localize individual collections of voxels that were correlated with gait metrics. The significant threshold was set at 0.001 to account for multiple comparisons among brain voxels. As this was an exploratory aim, no cluster-wise correction was applied. These secondary analyses enabled us to visually compare the location of voxel clusters and validated network-level relationships between functional connectivity and locomotor control relationships.

## Results

**FIGURE 3:**
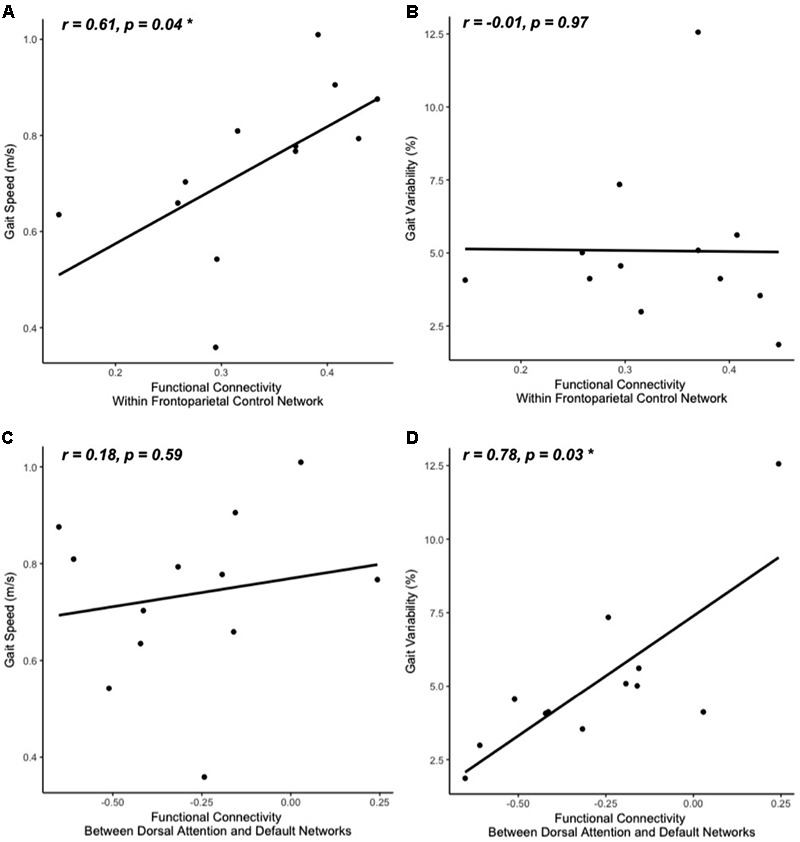
Gait speed **(A,C)** and variability **(B,D)** correlations with resting-state functional connectivity within cognitive networks: frontoparietal control **(A,B)** and default-dorsal attention **(C,D)**. Participants who walked with greater gait speed tended to have stronger functional connectivity *within* the frontoparietal control network **(A)**. Those who exhibited less stride time variability tended to have stronger functional connectivity *between* the dorsal attention network and the default network (greater negative values reflect stronger anti-phase connectivity, **D**). No other correlations between walking metrics and resting-state network connectivity reached significance (**B,C**; all other correlations not pictured).

The clinical characteristics of study participants were summarized in **Table 1**. The spatial topography of the network organization of the frontoparietal control network (**Figure 1**) and default network-dorsal attention network “anticorrelation” (**Figure 2**) showed similarity to previously described spatial organization of these networks when observed in healthy participants (Fox et al., 2005; Vincent et al., 2008; Yeo et al., 2011). Gait speed (Mean ± SD: 0.74 ± 0.17 m/s) and gait variability (Mean ± SD: 5.07 ± 2.72%) were not significantly correlated with one another (*R = -0.24, p = 0.45*). These metrics were correlated with the strength of functional connectivity within or between unique brain networks (**Figure 3**). Specifically, those with faster gait speed had stronger functional connectivity *within* the frontoparietal control network (*R = 0.61, p = 0.04*, **Figure 3A**). This relationship was independent of age (adjusted *p = 0.05)*. Gait speed was not significantly correlated with functional connectivity either within or between any other brain networks. In contrast, those with steadier gait (i.e., less stride time variability) exhibited stronger *negative* functional connectivity *between* the dorsal attention network and the default network (*R = 0.78, p < 0.01*, **Figure 3D**). In other words, less variable gait was linked to a greater degree of anti-phase correlation in BOLD signals between these two networks. This relationship also remained significant after adjusting for age (adjusted *p < 0.01).* Gait variability was not correlated with the strength of functional connectivity *within* the frontoparietal control network (*p = 0.97*, **Figure 3B**) or any other analyzed within- or between- network connectivity measure.

**Table 1:** Clinical characteristics of study participants.

Measure	Mean ±*SD*	Range
Age (y/o)	76.2 ± 9.5	66–93
4-Meter Walk Test (m/s)	0.7 ± 0.2	0.46–0.99
TMT – part A (sec)	66.0 ± 32.0	23.9–139.2
TMT – part B (sec)	247.5 ± 118.6	97.3–300.0
MMSE (pts)	25.3 ± 3.2	19–29
GDS (pts)	3.8 ± 3.1	0–9
Systolic BP (mmHg)	150.4 ± 21.5	116.0–178.0
Diastolic BP (mmHg)	71.5 ± 10.8	65.5–80.5

*TMT, Trail Making Test; MMSE, Mini-Mental State Examination; GDS, Geriatric Depression Scale; BP, blood pressure.*

**FIGURE 4:**
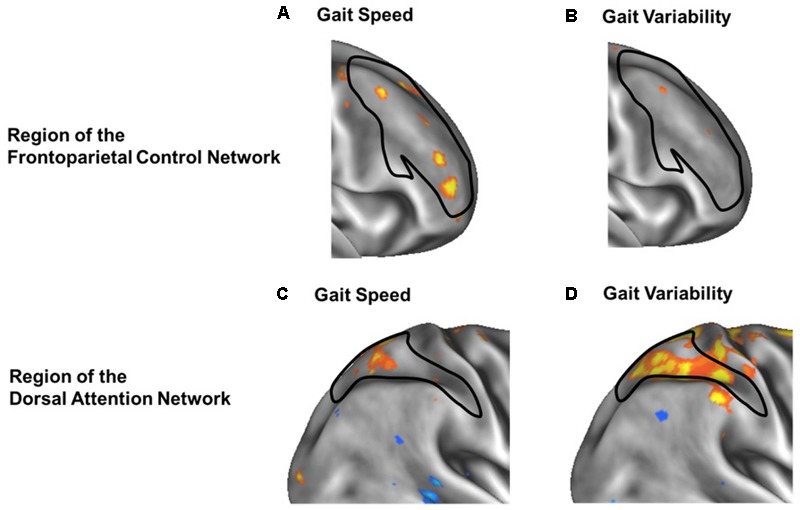
Voxel-wise analysis of within-network **(A,B)** and between-network **(C,D)** correlation of functional connectivity with gait speed **(A,C)** or gait variability **(B,D)**. In each panel, warmer colors represent voxels whose strength of functional connectivity correlated with gait speed or variability, when seeding either the frontoparietal control network **(A,B)** or the default network **(C,D)**. Black outlines indicate the within-network regions of the frontoparietal control network **(A,B)** or dorsal attention network **(C,D)**. Within frontoparietal control network, voxelwise functional connectivity is more strongly associated with gait speed **(A)** than gait variability **(B)**. In contrast, between-network functional connectivity from the default network to the dorsal attention network (black outline) is less strongly associated with gait speed **(C)** but more strongly associated with gait variability **(D)**.

Secondary voxel-wise analyses identified the locations where the strength of functional connectivity correlated with gait speed or variability, confirming a spatial organization consistient with increases within network for gait speed and between-network for gait variability. Several regions within the frontoparietal control network correlated with gait speed were found primarily located within the bilateral middle frontal gyrus [MNI coordinates: +39, +44, +11; -41, +42, 0; -40, +21, +23] (**Figures 4A,C**). In contrast, a between-network voxelwise correlation was found in the dorsal attention network, when correlating the strength of voxels connectivity with the default network functional connectivity against gait variability (**Figures 4B,D**). This region was located within the right superior parietal sulcus [MNI coordinates: +30, -48, +53]. None of these clusters remained significant after adjusting for multiple comparisons. However, their locations help confirm that the aforementioned relationships between functional connectivity and gait metrics *at the network-level* stemmed from more focal, voxel-level relationships within the larger functional networks.

## Discussion

This study examined the relationship among two common metrics of locomotor control and the functional connectivity of established large-scale brain networks in older adults with slow gait and executive dysfunction. The results suggest that gait speed and variability were associated with separate functional brain networks: gait speed was correlated with the strength of functional connectivity within the frontoparietal control network, whereas gait stride time variability was correlated with the strength of anti-phase functional connectivity between the dorsal attention network and the default network. Exploratory voxel-wise analyses further suggest that gait speed was specifically linked to the functional connectivity of the bilateral middle frontal gyri within the frontoparietal control network. Gait variability, on the other hand, was primarily linked to the right superior parietal sulcus within the dorsal attention network.

The frontoparietal control network is critically involved in executive function (Alvarez and Emory, 2006; Damoiseaux et al., 2006; Reineberg et al., 2015). Executive function is an umbrella term for a series of cognitive processes that give rise to goal-oriented behavior such as response inhibition, shifting of attention and working memory (Testa et al., 2012; Reineberg et al., 2015; Reineberg and Banich, 2016). Each of these subcomponents of executive function is governed by specific brain regions within and beyond the frontoparietal control network (Miyake et al., 2000; Miller and Cohen, 2001; Alvarez and Emory, 2006; Reineberg et al., 2015; Connolly et al., 2016; Reineberg and Banich, 2016). Our observation that those with faster gait speed have stronger functional connectivity *within* the frontoparietal control network—and particularly the middle frontal gyri—suggests that this widely assessed characteristic of locomotor control depends upon the integrity of communication within a collection of brain regions linked to executive function. This result is supported by previous studies (Yuan et al., 2015; Jor’dan et al., 2017) that gait speed was correlated with functional connectivity of the frontoparietal control network. However, their works either did not specify the strength or direction of this *gait speed – brain network* relationship (Yuan et al., 2015) or did not investigate the link between gait variability and functional brain networks (Jor’dan et al., 2017). Our results strengthen the notion that gait speed is dependent upon the integrity of the frontal control network. Furthermore, we found that gait variability is correlated with the between-network functional connectivity.

The degree of anti-phase functional connectivity between the dorsal attention and default networks has been linked to one’s ability to allocate attention to a given task and sustain it over time (Esterman et al., 2014; Dixon et al., 2017). Specifically, individuals with stronger anti-phase activity between these networks, as measured during rest, tend to exhibit less “intra-individual variability” in behavioral performance; that is, they have less variation in reaction time to a congruent or incongruent stimulus when presented visually at random time intervals. This outcome is considered an index for how efficiently one is able to allocate and sustain attentional resources (Bellgrove et al., 2004; Kelly et al., 2008) and exaggerated intra-individual variability is often viewed as a hallmark of attentional impairments (West et al., 2002; Kelly et al., 2008; Duchek et al., 2009). Our observations indicate that those with stronger anti-correlated resting brain activity between the dorsal attention network and the default network have less gait variability. Together, these results suggest that gait variability (or steadiness) is at least in part reliant upon one’s ability to sustain their attention over time, and at the physiologic level, dissociate the activity of these two networks. This notion is supported by previous studies demonstrating that as compared to walking under normal conditions, walking while simultaneously completing an attention-demanding task (e.g., mental arithmetic) increases gait variability, especially in those individuals with Parkinson’s disease (Hausdorff et al., 2003; Yogev et al., 2005) or Alzheimer’s disease (Sheridan et al., 2003).

While the current preliminary results provide proof-of-principle that gait speed and gait variability are linked to different brain networks, the small sample size limited our ability to identify more specific brain regions associated with gait metrics. Moreover, participants in this study presented with a common aging phenotype of relatively slow gait and mild-to-moderate cognitive impairment, yet did not suffer from dementia or other major neurological or musculoskeletal disorder. Future studies are warranted with larger sample sizes and a wider range of clinical populations to further identify and delineate relationships between functional connectivity and gait. As hypertension may affect resting-state functional connectivity in older adults (D’Esposito et al., 2003; Son et al., 2015), future studies are needed to examine the role of this and other cardiovascular and cerebrovascular outcomes on the current observed relationships. Moreover, in this study, we only examined gait during walking under normal, quiet conditions. Future studies should thus investigate the relationships between one’s ability to walk while performing a cognitive “dual” task, as related gait outcomes provide an accurate estimation of cognitive excitability (Hobert et al., 2017) and have been linked to future falls and dementia in older adults.

## Author Contributions

O-YL: Analysis and interpretation of the data, wrote the manuscript. MH: Analysis of data, critical revision of the manuscript for important intellectual content. JZ and LL: Critical revisions of the manuscript for important intellectual content. RH: Acquisition of data. BM: Study concept and design, study supervision, critical revisions of the manuscript for important intellectual content.

## Conflict of Interest Statement

The authors declare that the research was conducted in the absence of any commercial or financial relationships that could be construed as a potential conflict of interest.

## References

[B1] Alvarez J. A. Emory E. (2006). Executive function and the frontal lobes: a meta-analytic review. *Neuropsychol. Rev.* 16 17–42. 10.1007/s11065-006-9002-x 16794878

[B2] Arbuthnott K. Frank J. (2000). Trail making test, part B as a measure of executive control: validation using a set-switching paradigm. *J. Clin. Exp. Neuropsychol.* 22 518–528. 10.1076/1380-3395(200008)22:4;1-0;FT518 10923061

[B3] Balasubramanian C. K. Neptune R. R. Kautz S. A. (2009). Variability in spatiotemporal step characteristics and its relationship to walking performance post-stroke. *Gait Posture* 29 408–414. 10.1016/j.gaitpost.2008.10.061 19056272 PMC2675553

[B4] Bellgrove M. A. Hester R. Garavan H. (2004). The functional neuroanatomical correlates of response variability: evidence from a response inhibition task. *Neuropsychologia* 42 1910–1916. 10.1016/j.neuropsychologia.2004.05.007 15381021

[B5] Bilney B. Morris M. Webster K. (2003). Concurrent related validity of the GAITRite walkway system for quantification of the spatial and temporal parameters of gait. *Gait Posture* 17 68–74. 12535728 10.1016/s0966-6362(02)00053-x

[B6] Biswal B. Yetkin F. Z. Haughton V. M. Hyde J. S. (1995). Functional connectivity in the motor cortex of resting human brain using echo-planar MRI. *Magn. Reson. Med.* 34 537–541. 8524021 10.1002/mrm.1910340409

[B7] Brach J. S. Berthold R. Craik R. VanSwearingen J. M. Newman A. B. (2001). Gait variability in community-dwelling older adults. *J. Am. Geriatr. Soc.* 49 1646–1650. 11843998 10.1046/j.1532-5415.2001.t01-1-49274.x

[B8] Brach J. S. Perera S. Studenski S. Newman A. B. (2008). The reliability and validity of measures of gait variability in community-dwelling older adults. *Arch. Phys. Med. Rehabil.* 89 2293–2296. 10.1016/j.apmr.2008.06.010 19061741 PMC2705958

[B9] Connolly J. McNulty J. P. Boran L. Roche R. A. P. Delany D. Bokde A. L. W. (2016). Identification of resting state networks involved in executive function. *Brain Connect.* 6 365–374. 10.1089/brain.2015.0399 26935902

[B10] Cruz-Gómez Á. J. Ventura-Campos N. Belenguer A. Ávila C. Forn C. (2014). The link between resting-state functional connectivity and cognition in MS patients. *Mult. Scler.* 20 338–348. 10.1177/1352458513495584 23828871

[B11] Damoiseaux J. S. Rombouts S. A. R. B. Barkhof F. Scheltens P. Stam C. J. Smith S. M. (2006). Consistent resting-state networks across healthy subjects. *Proc. Natl. Acad. Sci. U.S.A.* 103 13848–13853. 10.1073/pnas.0601417103 16945915 PMC1564249

[B12] D’Esposito M. Deouell L. Y. Gazzaley A. (2003). Alterations in the BOLD fMRI signal with ageing and disease: a challenge for neuroimaging. *Nat. Rev. Neurosci.* 4 863–872. 10.1038/nrn1246 14595398

[B13] Dixon M. L. Andrews-Hanna J. R. Spreng R. N. Irving Z. C. Mills C. Girn M. (2017). Interactions between the default network and dorsal attention network vary across default subsystems, time, and cognitive states. *Neuroimage* 147 632–649. 10.1016/j.neuroimage.2016.12.073 28040543

[B14] Duchek J. M. Balota D. A. Tse C.-S. Holtzman D. M. Fagan A. M. Goate A. M. (2009). The utility of intraindividual variability in selective attention tasks as an early marker for Alzheimer’s disease. *Neuropsychology* 23 746–758. 10.1037/a0016583 19899833 PMC2779520

[B15] Eldaief M. C. Halko M. A. Buckner R. L. Pascual-Leone A. (2011). Transcranial magnetic stimulation modulates the brain’s intrinsic activity in a frequency-dependent manner. *Proc. Natl. Acad. Sci. U.S.A.* 108 21229–21234. 10.1073/pnas.1113103109 22160708 PMC3248528

[B16] Esterman M. Rosenberg M. D. Noonan S. K. (2014). Intrinsic fluctuations in sustained attention and distractor processing. *J. Neurosci.* 34 1724–1730. 10.1523/JNEUROSCI.2658-13.2014 24478354 PMC6827583

[B17] Folstein M. F. Folstein S. E. McHugh P. R. (1975). “Mini-mental state”. A practical method for grading the cognitive state of patients for the clinician. *J Psychiatr. Res.* 12 189–198. 1202204 10.1016/0022-3956(75)90026-6

[B18] Fox M. D. Raichle M. E. (2007). Spontaneous fluctuations in brain activity observed with functional magnetic resonance imaging. *Nat. Rev. Neurosci.* 8 700–711. 10.1038/nrn2201 17704812

[B19] Fox M. D. Snyder A. Z. Vincent J. L. Corbetta M. Van Essen D. C. Raichle M. E. (2005). The human brain is intrinsically organized into dynamic, anticorrelated functional networks. *Proc. Natl. Acad. Sci. U.S.A.* 102 9673–9678. 10.1073/pnas.0504136102 15976020 PMC1157105

[B20] Guralnik J. M. Ferrucci L. Simonsick E. M. Salive M. E. Wallace R. B. (1995). Lower-extremity function in persons over the age of 70 years as a predictor of subsequent disability. *N. Engl. J. Med.* 332 556–561. 10.1056/NEJM199503023320902 7838189 PMC9828188

[B21] Halko M. A. Farzan F. Eldaief M. C. Schmahmann J. D. Pascual-Leone A. (2014). Intermittent theta-burst stimulation of the lateral cerebellum increases functional connectivity of the default network. *J. Neurosci.* 34 12049–12056. 10.1523/JNEUROSCI.1776-14.2014 25186750 PMC4152606

[B22] Hamacher D. Herold F. Wiegel P. Hamacher D. Schega L. (2015). Brain activity during walking: a systematic review. *Neurosci. Biobehav. Rev.* 57 310–327. 10.1016/j.neubiorev.2015.08.002 26306029

[B23] Hausdorff J. M. (2005). Gait variability: methods, modeling and meaning. *J. Neuroeng. Rehabil.* 2:19. 10.1186/1743-0003-2-19 16033650 PMC1185560

[B24] Hausdorff J. M. (2007). Gait dynamics, fractals and falls: finding meaning in the stride-to-stride fluctuations of human walking. *Hum. Mov. Sci.* 26 555–589. 10.1016/j.humov.2007.05.003 17618701 PMC2267927

[B25] Hausdorff J. M. Balash J. Giladi N. (2003). Effects of cognitive challenge on gait variability in patients with Parkinson’s disease. *J. Geriatr. Psychiatry Neurol.* 16 53–58. 10.1177/0891988702250580 12641374

[B26] Hausdorff J. M. Cudkowicz M. E. Firtion R. Wei J. Y. Goldberger A. L. (1998). Gait variability and basal ganglia disorders: stride-to-stride variations of gait cycle timing in Parkinson’s disease and Huntington’s disease. *Mov. Disord.* 13 428–437. 10.1002/mds.870130310 9613733

[B27] Hausdorff J. M. Rios D. A. Edelberg H. K. (2001). Gait variability and fall risk in community-living older adults: a 1-year prospective study. *Arch. Phys. Med. Rehabil.* 82 1050–1056. 10.1053/apmr.2001.24893 11494184

[B28] Hobert M. A. Meyer S. I. Hasmann S. E. Metzger F. G. Suenkel U. Eschweiler G. W. (2017). Gait is associated with cognitive flexibility: a dual-tasking study in healthy older people. *Front. Aging Neurosci.* 9:154. 10.3389/fnagi.2017.00154 28596731 PMC5442228

[B29] Hollman J. H. Childs K. B. McNeil M. L. Mueller A. C. Quilter C. M. Youdas J. W. (2010). Number of strides required for reliable measurements of pace, rhythm and variability parameters of gait during normal and dual task walking in older individuals. *Gait Posture* 32 23–28. 10.1016/j.gaitpost.2010.02.017 20363136

[B30] Hollman J. H. McDade E. M. Petersen R. C. (2011). Normative spatiotemporal gait parameters in older adults. *Gait Posture* 34 111–118. 10.1016/j.gaitpost.2011.03.024 21531139 PMC3104090

[B31] Jor’dan A. J. Poole V. N. Iloputaife I. Milberg W. Manor B. Esterman M. (2017). Executive network activation is linked to walking speed in older adults: functional MRI and TCD ultrasound evidence from the MOBILIZE Boston study. *J. Gerontol. A Biol. Sci. Med. Sci.* 72 1669–1675. 10.1093/gerona/glx063 28449077 PMC5861979

[B32] Kelly A. M. C. Uddin L. Q. Biswal B. B. Castellanos F. X. Milham M. P. (2008). Competition between functional brain networks mediates behavioral variability. *Neuroimage* 39 527–537. 10.1016/j.neuroimage.2007.08.008 17919929

[B33] Kroneberg D. Elshehabi M. Meyer A.-C. Doss S. Kühn A. Maetzler W. (2017). How many steps are enough? Assessment of gait variability in realistically confined clinical settings. *Basal Ganglia* 8 3–4. 10.1016/j.baga.2017.02.009

[B34] Lee M. H. Smyser C. D. Shimony J. S. (2013). Resting-state fMRI: a review of methods and clinical applications. *Am. J. Neuroradiol.* 34 1866–1872. 10.3174/ajnr.A3263 22936095 PMC4035703

[B35] Lord S. Galna B. Verghese J. Coleman S. Burn D. Rochester L. (2013). Independent domains of gait in older adults and associated motor and nonmotor attributes: validation of a factor analysis approach. *J. Gerontol. A Biol. Sci. Med. Sci.* 68 820–827. 10.1093/gerona/gls255 23250001

[B36] McDonough A. L. Batavia M. Chen F. C. Kwon S. Ziai J. (2001). The validity and reliability of the GAITRite system’s measurements: a preliminary evaluation. *Arch. Phys. Med. Rehabil.* 82 419–425. 10.1053/apmr.2001.19778 11245768

[B37] Miller E. K. Cohen J. D. (2001). An integrative theory of prefrontal cortex function. *Annu. Rev. Neurosci.* 24 167–202. 10.1146/annurev.neuro.24.1.167 11283309

[B38] Miyake A. Friedman N. P. Emerson M. J. Witzki A. H. Howerter A. Wager T. D. (2000). The unity and diversity of executive functions and their contributions to complex “Frontal Lobe” tasks: a latent variable analysis. *Cogn. Psychol.* 41 49–100. 10.1006/cogp.1999.0734 10945922

[B39] Perera S. Smith C. Coffman L. Brach J. (2016). Number of steps needed for reliable gait variability measurement. *Gerontologist* 56 335–336. 10.1093/geront/gnw162.1366 24829307

[B40] Reineberg A. E. Andrews-Hanna J. R. Depue B. E. Friedman N. P. Banich M. T. (2015). Resting-state networks predict individual differences in common and specific aspects of executive function. *Neuroimage* 104 69–78. 10.1016/j.neuroimage.2014.09.045 25281800 PMC4262251

[B41] Reineberg A. E. Banich M. T. (2016). Functional connectivity at rest is sensitive to individual differences in executive function: a network analysis. *Hum. Brain Mapp.* 37 2959–2975. 10.1002/hbm.23219 27167614 PMC6186291

[B42] Sheridan P. L. Solomont J. Kowall N. Hausdorff J. M. (2003). Influence of executive function on locomotor function: divided attention increases gait variability in Alzheimer’s disease. *J. Am. Geriatr. Soc.* 51 1633–1637. 14687395 10.1046/j.1532-5415.2003.51516.x

[B43] Son S. J. Kim J. Lee E. Park J. Y. Namkoong K. Hong C. H. (2015). Effect of hypertension on the resting-state functional connectivity in patients with Alzheimer’s disease (AD). *Arch. Gerontol. Geriatr.* 60 210–216. 10.1016/j.archger.2014.09.012 25307953

[B44] Testa R. Bennett P. Ponsford J. (2012). Factor analysis of nineteen executive function tests in a healthy adult population. *Arch. Clin. Neuropsychol.* 27 213–224. 10.1093/arclin/acr112 22314610

[B45] Tombaugh T. N. (2004). Trail Making Test A and B: normative data stratified by age and education. *Arch. Clin. Neuropsychol.* 19 203–214. 10.1016/S0887-6177(03)00039-8 15010086

[B46] Tombaugh T. N. McIntyre N. J. (1992). The mini-mental state examination: a comprehensive review. *J. Am. Geriatr. Soc.* 40 922–935. 1512391 10.1111/j.1532-5415.1992.tb01992.x

[B47] van den Heuvel M. P. Pol H. E. H. (2010). Exploring the brain network: a review on resting-state fMRI functional connectivity. *Eur. Neuropsychopharmacol.* 20 519–534. 10.1016/j.euroneuro.2010.03.008 20471808

[B48] van Marwijk H. W. Wallace P. de Bock G. H. Hermans J. Kaptein A. A. Mulder J. D. (1995). Evaluation of the feasibility, reliability and diagnostic value of shortened versions of the geriatric depression scale. *Br. J. Gen. Pract.* 45 195–199. 7612321 PMC1239201

[B49] Vincent J. L. Kahn I. Snyder A. Z. Raichle M. E. Buckner R. L. (2008). Evidence for a frontoparietal control system revealed by intrinsic functional connectivity. *J. Neurophysiol.* 100 3328–3342. 10.1152/jn.90355.2008 18799601 PMC2604839

[B50] West R. Murphy K. J. Armilio M. L. Craik F. I. M. Stuss D. T. (2002). Lapses of intention and performance variability reveal age-related increases in fluctuations of executive control. *Brain Cogn.* 49 402–419. 12139961 10.1006/brcg.2001.1507

[B51] Wittenberg E. Thompson J. Nam C. S. Franz J. R. (2017). Neuroimaging of human balance control: a systematic review. *Front. Hum. Neurosci.* 11:170. 10.3389/fnhum.2017.00170 28443007 PMC5385364

[B52] Yeo B. T. T. Krienen F. M. Sepulcre J. Sabuncu M. R. Lashkari D. Hollinshead M. (2011). The organization of the human cerebral cortex estimated by intrinsic functional connectivity. *J. Neurophysiol.* 106 1125–1165. 10.1152/jn.00338.2011 21653723 PMC3174820

[B53] Yogev G. Giladi N. Peretz C. Springer S. Simon E. S. Hausdorff J. M. (2005). Dual tasking, gait rhythmicity, and Parkinson’s disease: which aspects of gait are attention demanding? *Eur. J. Neurosci.* 22 1248–1256. 10.1111/j.1460-9568.2005.04298.x 16176368

[B54] Yuan J. Blumen H. M. Verghese J. Holtzer R. (2015). Functional connectivity associated with gait velocity during walking and walking-while-talking in aging: a resting-state fMRI study. *Hum. Brain Mapp.* 36 1484–1493. 10.1002/hbm.22717 25504964 PMC4373975

